# Liquid–liquid phase separation of tau protein: The crucial role of electrostatic interactions

**DOI:** 10.1074/jbc.AC119.009198

**Published:** 2019-05-16

**Authors:** Solomiia Boyko, Xu Qi, Tien-Hao Chen, Krystyna Surewicz, Witold K. Surewicz

**Affiliations:** Department of Physiology and Biophysics, Case Western Reserve University, Cleveland, Ohio 44106

**Keywords:** Tau protein (Tau), protein misfolding, tauopathy, neurodegenerative disease, protein-protein interaction, intrinsically disordered protein, liquid-liquid phase separation (LLPS), Alzheimer's disease, neurodegeneration, protein aggregation

## Abstract

Recent studies have indicated that tau, a protein involved in Alzheimer's disease and other neurodegenerative disorders, has a propensity to undergo liquid–liquid phase separation (LLPS). However, the mechanism of this process remains unknown. Here, we demonstrate that tau LLPS is largely driven by intermolecular electrostatic interactions between the negatively charged N-terminal and positively charged middle/C-terminal regions, whereas hydrophobic interactions play a surprisingly small role. Furthermore, our results reveal that, in contrast to previous suggestions, phosphorylation is not required for tau LLPS. These findings provide a foundation for understanding the mechanism by which phosphorylation and other posttranslational modifications could modulate tau LLPS in the context of specific physiological functions as well as pathological interactions.

## Introduction

Tau belongs to the family of microtubule-associated proteins (1, 2). Due to alternative splicing of the *MAPT* gene, tau in the human brain is expressed as six major isoforms, the longest of which, tau441, consists of two N-terminal inserts, a proline-rich region, and four imperfect 31–32-residue repeat sequences flanked by the C-terminal region (1, 2). The entire N-terminal part (including the Pro-rich region) is intrinsically disordered, whereas some local secondary structure exists within the repeat region (1). The shorter isoforms differ with respect to the number of N-terminal inserts and contain either three or four repeats. Whereas normally tau is in equilibrium between the free and microtubule-associated forms, under some conditions it aggregates into neurofibrillary tangles and other types of intracellular inclusions, and these aggregates are believed to play a key role in the pathogenesis of Alzheimer's disease and several other neurodegenerative disorders (2–4).

Spatial organization of cells typically revolves around membrane-bound organelles such as the nucleus, Golgi, or endoplasmic reticulum. However, rapidly growing evidence indicates that spatial segregation can also be accomplished by liquid demixing, whereby liquid droplets arise through liquid–liquid phase separation (LLPS)^4^ (5–9). Formation of these droplets is commonly associated with low-complexity protein sequences that remain natively unstructured, which permits a diversity of multivalent protein–protein and protein–nucleotide interactions (5–9). Biomolecular condensates formed via liquid–liquid phase transitions appear to be key for organizing the contents of living cells, playing an important role in numerous biological and pathophysiological processes (5–10).

Recently, it was reported that, akin to many other natively unstructured proteins (5–9), tau has a propensity to undergo LLPS (11–16). This was first observed upon mixing of tau with RNA (12) and, subsequently, for tau alone in the presence of crowding agents (11, 13). LLPS is likely to be of major consequences for pathological misfolding of tau, as the environment of liquid droplets has been shown to be conducive to aggregation/fibrillation of several other proteins involved in neurodegenerative diseases, including FUS, hnRNPA1, and TDP-43 (9, 10, 17–19). However, some of the reports regarding tau LLPS are controversial, and the mechanism by which the protein forms liquid droplets remains largely unknown. Here we demonstrate that, contrary to previous suggestions (11), liquid demixing of tau does not require phosphorylation. We also provide fundamental mechanistic insight into this process, revealing that tau LLPS is driven by attractive electrostatic intermolecular interactions between the negatively charged N-terminal and positively charged middle/C-terminal domains of the protein, with hydrophobic interactions playing a surprisingly small role.

## Results

**Figure 1.**
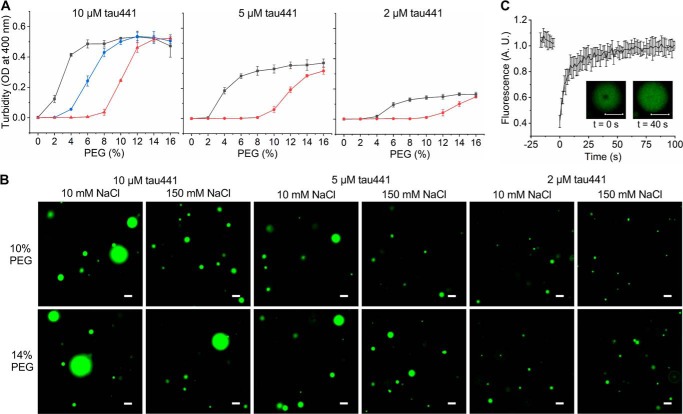
**LLPS of tau441.** *A*, LLPS at different tau441 concentrations as monitored by turbidity (OD at 400 nm) at 37 °C as a function of ionic strength (NaCl concentration) and concentration of the crowding agent, PEG 10,000. *Black*, 10 mm NaCl; *blue*, 75 mm NaCl; *red*, 150 mm NaCl. *Error bars*, S.D. (*n* = 5). *B*, representative fluorescence microscopy images of tau441 under different experimental conditions. The images were obtained at room temperature using a 1:20 ratio of Alexa fluor 488–labeled to unlabeled protein. *Scale bar*, 3 μm. *C*, representative FRAP data obtained within 10 min after droplet formation by the addition of 10% PEG. *Inset*, images of droplets 0 and 40 s after photobleaching (*scale bars*, 5 μm). *A.U.*, arbitrary units.

Freshly prepared solutions of recombinant full-length tau (tau441) in HEPES buffer (pH 7.4) at a wide range of protein concentrations (2–100 μm) show no measurable turbidity, indicating the presence of a protein (likely monomeric) in a single phase. However, upon the addition to tau441 of polyethylene glycol (PEG), the volume-excluding polymer frequently used to mimic intracellular crowding, we observed a rapid increase in sample turbidity, strongly suggesting LLPS (Fig. 1*A*). This turbidity increase was clearly detectable at a protein concentration as low as 2 μm. Importantly, under all conditions tested, the effect showed a strong dependence on the ionic strength of the buffer, with higher amounts of PEG required to induce LLPS in the presence of increasing concentrations of NaCl (Fig. 1*A*). For example, at a protein concentration of 10 μm, in a buffer containing 10 mm NaCl, the increase in turbidity to the *A*_400_ value of 0.1 was observed in the presence of as little as 2% PEG, whereas ∼5 and 9% PEG was required to produce a similar effect in a buffer containing 75 and 150 mm NaCl, respectively. The effect of ionic strength on tau441 LLPS is not salt-specific, as a similar behavior was observed using KCl as a monovalent salt (Fig. S1).

Because the increase in sample turbidity could also result from other effects (*e.g.* protein aggregation), the partitioning of tau441 into spherical droplets was directly confirmed by fluorescence microscopy using Alexa fluor 488–labeled tau441 (Fig. 1*B*). Consistent with turbidity data, droplets formed in the presence of 150 mm NaCl appeared generally smaller in size as compared with those in the presence of 10 mm NaCl. Furthermore, the size of droplets increased when the concentration of PEG was increased (Fig. 1*B*).

Material properties of freshly formed spherical particles (within ∼10 min after the addition of PEG) were characterized by fluorescence recovery after photobleaching (FRAP). These experiments revealed rapid recovery of the fluorescence signal, indicating fast protein diffusion and further confirming dynamic properties of the droplets. Collectively, these data clearly demonstrate that tau441 in the presence of a crowding agent undergoes LLPS, and this reaction is especially robust at lower ionic strengths. Importantly, the conditions under which we observe LLPS *in vitro* appear to be physiologically relevant, as conservative estimates of intracellular tau concentration are between ∼2 and 7 μm (11), and 10–15% PEG is within the range of concentrations typically used to mimic intracellular molecular crowding (20).

**Figure 2.**
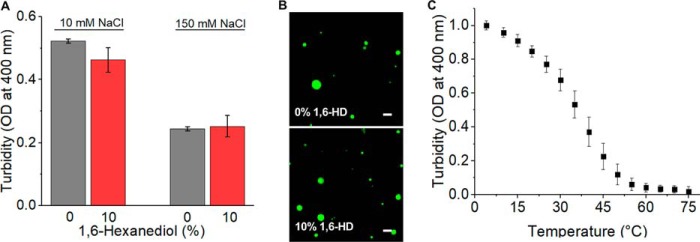
**The effect of 1,6-hexanediol (*1,6-HD*) and temperature on LLPS of tau441.** *A*, turbidity in the absence (*gray*) and presence (*red*) of 10% (v/w) 1,6-hexanediol. Experiments were performed at 37 °C in the presence of 10% PEG. *Error bars*, S.D. (*n* = 5). *B*, representative fluorescence microscopy images recorded at room temperature in a buffer containing 150 mm NaCl and 10% PEG in the absence (*top*) and presence of 10% 1,6-hexanediol (*bottom*). *Scale bar*, 3 μm. *C*, turbidity of tau411 (10 μm) in the presence of PEG (10%) as a function of temperature. Samples were prepared by rapidly mixing protein and PEG solutions preheated to 75 °C, and turbidity was monitored in a cooling cycle by decreasing temperature at a rate of 2.5 degrees/min. *Error bars*, S.D. (*n* = 4).

The decreased tendency of tau441 to form liquid droplets at increasing salt concentrations strongly suggests that LLPS is at least partly driven by attractive electrostatic interactions. To explore potential involvement of other types of interactions in this process, we employed 1,6-hexanediol, a compound known to inhibit formation of P granules and stress granules *in vivo* (21) as well as LLPS of proteins such as hnRNPA1 (17) or TDP-43 (19, 22) *in vitro*, presumably by disrupting hydrophobic interactions (23), even though the precise mechanism of this inhibition is unknown. Surprisingly, even 10% hexanediol had very little, if any, effect on tau441 turbidity in the presence of PEG (Fig. 2*A*). Furthermore, droplets in the presence and absence of 10% hexanediol appeared morphologically very similar (Fig. 2*B*). This contrasts with the behavior of hnRNPA1 or TDP-43, in which cases as little as 5% hexanediol is sufficient to completely abrogate LLPS (17, 19, 22). Thus, hydrophobic interactions appear to play a minor role in LLPS of tau441.

Another factor known to control liquid demixing of proteins (or protein/RNA mixtures) is temperature. Whereas classical LLPS-prone proteins such as FUS or hnRNPA1 exhibit the “upper critical solution temperature” (UCST) behavior, whereby transition to a two-phase system occurs only upon cooling below a certain critical temperature (17, 24), there are examples of proteins with “lower critical solution temperature” (LCST) transition, which occurs upon heating above a critical temperature (25, 26). Recent studies indicate that these two distinct types of transitions are encoded by the presence of specific amino acid sequence motifs in intrinsically disordered proteins (25, 26). LLPS of tau441 (in the presence of 150 mm NaCl) shows UCST-like behavior as judged by temperature dependence of turbidity (Fig. 2*C*). Even though full understanding of this UCST-like behavior of tau441 will require further studies, this behavior may be related to the presence of positively and negatively charged domains (see below), as electrostatic interactions between oppositely charged patches have been proposed to contribute enthalpically to UCST phase transitions (26). Interestingly, the UCST behavior of tau alone is dramatically different from that of tau mixture with RNA, in which case rapid transition to a two-phase system was observed upon sample heating above 22 °C (12, 16).

**Figure 3.**
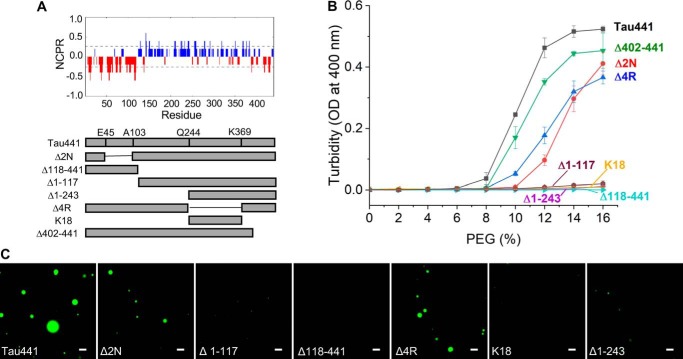
**The effect of truncations and deletions on the propensity of tau441 to undergo LLPS.** *A*, *top*, net charge per residue (*NCPR*) plot for tau441 at neutral pH generated using the algorithm available on the CIDER (33) webserver with a five-residue window. *Bottom*, *schematic diagram* of tau441 deletion/truncation variants used in this study. *B*, turbidity of individual tau variants (10 μm in each case) as a function of PEG concentration. *Error bars*, S.D. (*n* = 3). *C*, representative fluorescence microscopy images for different tau variants (10 μm in each case) in the presence of 12% PEG. *Scale bars*, 3 μm.

Analysis of charge distribution in tau441 reveals a high degree of polarization, with the N-terminal region up to residue ∼117 being negatively charged and the middle and most of the C-terminal parts of the molecule (residues 118–402) being positively charged (Fig. 3*A*, *top*). This, together with data described above, led us to hypothesize that LLPS of tau441 is largely driven by attractive electrostatic intermolecular interactions between the negatively charged N-terminal and positively charged middle/C-terminal regions. To test this hypothesis and assess the role of individual tau441 domains in LLPS, we expressed and purified a number of deletion variants of tau441 (Fig. 3*A*, *bottom*). First, we tested the physiologically important Δ2N variant in which the two N-terminal inserts (residues 45–103) were missing. This deletion reduces the length of the negatively charged region within the N-terminal part of the protein. Consistent with our hypothesis, the propensity of Δ2N tau to undergo LLPS was diminished, as indicated by lower turbidity and the requirement for higher concentrations of the crowding agent as compared with that for tau441 (Fig. 3*B*). However, droplets still could be observed for this variant by microscopy, especially at higher concentrations of PEG (Fig. 3*C*). Building on this finding, next we performed experiments with the N-truncated variant tauΔ1–117, in which the entire negatively charged segment had been removed. This deletion resulted in an essentially complete loss of the ability to undergo LLPS (Fig. 3, *B* and *C*), further reinforcing our central hypothesis regarding the crucial role of intermolecular electrostatic interactions between oppositely charged regions of tau. Consistent with this notion, no phase separation was observed for the negatively charged N-terminal fragment (tauΔ118–441) alone (Fig. 3, *B* and *C*).

**Figure 4.**
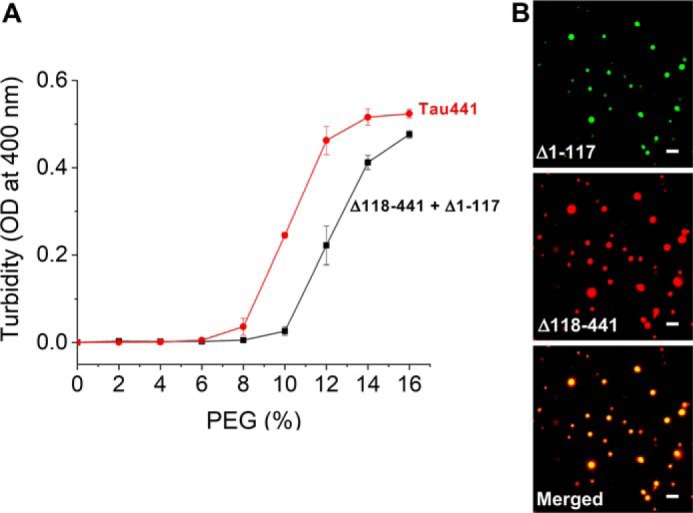
**The mixture of tauΔ1–117 and tauΔ118–441 undergoes LLPS.** *A*, turbidity of the mixture of tauΔ1–117 and tauΔ118–441 (10 μm each) as a function of PEG concentration. Data for tau441 are shown for comparison. *Error bars*, S.D. (*n* = 3). *B*, representative fluorescence microscopy images showing co-localization of tauΔ1–117 with tauΔ118–441 in liquid droplets. The mixture of two proteins (10 μm each) contained Alexa fluor 488 (tauΔ1–117) and Alexa fluor 594 (tauΔ118–441) variants (5%). The images were obtained at room temperature in the presence of 150 mm NaCl and 12% PEG. *Scale bar*, 3 μm.

Deletion of the entire four-repeat (4R) region (residues 244–369) modestly decreased the propensity of tau to undergo LLPS, as indicated by reduced sample turbidity (Fig. 3*B*), but droplets still could be observed by microscopy (Fig. 3*C*). This suggests that the repeats, a region critical for pathological aggregation of tau (1–4), are not essential for LLPS. The latter notion is consistent with the finding that neither K18 (a polypeptide consisting of the repeat region alone) nor the longer fragment tauΔ1–243 (encompassing K18 and the C-terminal domain) has much propensity to form droplets under the present experimental conditions. However, phase separation was observed when the positively charged tauΔ1–117 and negatively charged tauΔ118–441 fragments (neither of which shows significant propensity for LLPS alone) were mixed together (Fig. 4), further supporting the contention that LLPS of tau is driven by attractive electrostatic interactions between the oppositely charged regions. Finally, the most C-terminal 402–441 segment that is predominantly negatively charged (especially within residues 418–434) appears to play a minor role, as deletion of this entire segment (tauΔ402–441) had little effect on LLPS (Fig. 3*B*).

It should be noted that a recent study reported droplet formation for the K18 fragment of tau (14). However, most experiments in the latter study were performed under nonphysiological conditions (protein concentration ∼100 μm, pH 8.8), and droplets could be observed only after very long incubation times (24–72 h), which is highly unusual for protein LLPS. Our experiments provide no evidence for significant LLPS of K18 under the experimental conditions used in the present study.

## Discussion

Previous studies on tau LLPS focused largely on protein derived from insect Sf9 cells (11, 13). The latter protein was phosphorylated (although with a phosphorylation pattern substantially different from that observed in Alzheimer's disease (27)), and the authors concluded that phosphorylation is a major driver (if not a prerequisite) for LLPS (11). The latter notion is not supported by the present study, as we clearly demonstrate that nonphosphorylated protein also undergoes robust LLPS under physiologically relevant conditions. Molecular-level interpretation of data for protein purified from Sf9 cells is less than straightforward, because this preparation likely contains multiple isomers with distinct populations of phosphorylated sites. Nevertheless, a remarkable finding of one of these studies (which was based solely on microscopic observations) was that LLPS of Sf9 cell–derived tau is inhibited by hexanediol and shows very high resistance to salts, suggesting a major role of hydrophobic interactions (11) (even though salt sensitivity was reported in another study with the same protein (13)). The findings for Sf9 cell–derived protein are in sharp contrast to our present data for nonphosphorylated tau, as the latter data clearly point to the key role in LLPS of attractive intermolecular electrostatic interactions between oppositely charged regions, with little involvement of hydrophobic interactions. At this juncture, it is difficult to explain how tau phosphorylation *per se* could increase the strength of hydrophobic interactions. One way to reconcile those findings is the possibility that other posttranslational modifications could also contribute to LLPS behavior of tau expressed in Sf9 cells.

Another type of interactions of key importance for LLPS of some proteins are cation–π interactions among aromatic amino acids and basic residues (6, 9, 28) (especially tyrosine–arginine interactions, as recently shown for Fused in Sarcoma family proteins (29)) and π–π stacking interactions between aromatic side chains (6, 9, 28). These interactions are, however, less likely to be a major driving force for phase separation of tau, because the content of aromatic residues in this protein is unusually low (five tyrosines, three phenylalanines, and no tryptophans). Furthermore, deletion of the negatively charged N-terminal 1–117 segment that contains only three aromatic amino acids (two Tyr residues and one Phe residue) results in an essentially complete abrogation of LLPS.

Phosphorylation is known to affect both the functional state and pathological aggregation of tau (2, 30, 31). However, the pattern of phosphorylation and the specific sites involved are strongly dependent on the physiological context. Given that one of the main consequences of phosphorylation is charge alteration, the present insight regarding the key role of electrostatic interactions provides a foundation for rational design of future experiments to examine how phosphorylation of individual Ser/Thr residues in tau modulates LLPS in the context of specific physiological functions and/or pathological interactions.

## Experimental procedures

### Expression, purification, and labeling of tau variants

Constructs for all tau variants were subcloned into pET-15b vector between NdelI and EcoRI restriction sites. Proteins were expressed in *Escherichia coli* BL21 (DE3). Bacteria were grown at 37 °C and harvested 3 h after induction with 1 mm isopropyl 1-thio-β-d-galactopyranoside. Proteins without a poly-His tag were purified using a published protocol (32) with some modifications. Briefly, cell pellets were suspended in the lysis buffer (20 mm MES, 500 mm NaCl, 1 mm EDTA, 0.2 mm MgCl_2_, 5 mm DTT, 1 mm PMSF, protease inhibitor mixture, pH 6.8) and lysed by sonication on ice. Lysates were boiled for 20 min, cooled on ice, and centrifuged at 127,000 × *g* for 40 min to remove cell debris and aggregated proteins. Protein variants with basic pI (tau441, tauΔ2N, and tauΔ1–117) were dialyzed against buffer containing 20 mm MES, 50 mm NaCl, 1 mm EDTA, 1 mm MgCl_2_, 2 mm DTT, 0.1 mm PMSF, pH 6.8, purified on a cation-exchange column (SP Sepharose HP, GE Healthcare). The variant tauΔ4R was dialyzed against buffer B (10 mm potassium phosphate, 20 mm NaCl, 2 mm DTT, 0.1 mm PMSF, pH 8.5) and purified on an anion-exchange column (Mono-Q, GE Healthcare). In each case, proteins were eluted using a linear gradient of NaCl, and fractions containing tau were pooled, concentrated, and further purified using size-exclusion chromatography (Superdex 75 10/300 GL) in 10 mm HEPES buffer, pH 7.4, containing 100 mm NaCl and 2 mm DTT, 0.1 mm PMSF. Tau variants Δ1–243, Δ118–241, and K18 were expressed with the N-terminal His_6_ tag and a linker containing thrombin cleavage site. These proteins were purified on an immobilized nickel-affinity column (Ni-NTA Fastflow, Qiagen), followed by size-exclusion chromatography as described above. The His tag was removed by incubating for 2 h at room temperature with biotinylated thrombin (Novagen; 0.5 units/mg protein). Thrombin was then captured using streptavidin-agarose beads, and free His tag was removed by dialysis against 10 mm HEPES buffer, pH 7.4, containing 100 mm NaCl and 2 mm DTT, 0.1 mm PMSF. The cleaved proteins contained two additional N-terminal residues (GS). Protein purity was verified by gel electrophoresis, and the identity of individual variants was confirmed by electrospray MS. Protein concentration was determined using a reducing agent–compatible BCA protein assay (Thermo Fisher Scientific).

Proteins were labeled with Alexa fluor 488 or 594 (Invitrogen) by adding 10 μl of the dye in DMSO (10 mg/ml) to 100 μl of the protein (10 mg/ml) in 0.1 m sodium bicarbonate buffer, pH 8.3, and incubating the mixture at room temperature for 2 h with stirring. Excess dye was removed using Zeba spin desalting columns (Thermo Fisher Scientific). Labeling efficiency was estimated based on relative concentrations of the protein and the dye (the latter determined by absorbance at 495 or 490 nm).

### Monitoring of LLPS

Liquid demixing of tau variants was monitored by turbidity (optical density at 400 nm) at 37 °C in 10 mm HEPES buffer (pH 7.4) containing 1 mm DTT and NaCl and PEG 10,000 (Sigma-Aldrich) at appropriate concentrations. These measurements were performed using the M1000 Tecan plate reader. Temperature-dependent changes in turbidity were measured on a Cary 100 Bio spectrophotometer equipped with a Peltier temperature control unit. Droplets were visualized by fluorescence microscopy using a 1:20 molar ratio of Alexa fluor 488–labeled to unlabeled protein. To this end, samples (20 μl) were placed on the glass bottom of a 35-mm dish that was covered with a microscope coverglass and sealed to prevent evaporation. The measurements were performed within 5 min after mixing of the protein with PEG, and the images were obtained in solution away from the bottom of the dish. Microscopy experiments were performed on a Keyence BZ-X710 microscope with a ×100/1.45 numerical aperture oil-immersion lens.

### FRAP

FRAP was performed using a Leica HyVolution SP8 confocal microscope with 2.4-milliwatt laser intensity for bleaching, ×63/1.4 numerical aperture oil-immersion objective, and photomultiplier tube detector. In each experiment, three droplets of ∼5-μm diameter were selected, and the measurements involved 10 prebleaching frames, five flashes of bleaching (2.4-milliwatt laser power), and 100 post-bleaching frames (1.3 s/frame). Data were analyzed using Leica LAX suite.

## Author contributions

S. B. and W. K. S. conceptualization; S. B., X. Q., T.-H. C., K. S., and W. K. S. data curation; S. B., X. Q., T.-H. C., and K. S. formal analysis; S. B. and W. K. S. funding acquisition; S. B., X. Q., T.-H. C., K. S., and W. K. S. investigation; S. B. and W. K. S. writing-original draft; S. B. and W. K. S. project administration; S. B., X. Q., T.-H. C., K. S., and W. K. S. writing-review and editing.

## Supplementary Material

Supporting Information

## Footnotes

4 The abbreviations used are:

LLPS: liquid–liquid phase separation
FRAP: fluorescence recovery after photobleaching
UCST: upper critical solution temperature
LCST: lower critical solution temperature
PMSF: phenylmethylsulfonyl fluoride.
