# Supplementary material for: Liquid–liquid phase separation of tau protein: The crucial role of electrostatic interactions
Source: J Biol Chem. 2019 May 16;294(29):11054–9. doi: 10.1074/jbc.AC119.009198 (PMC6643045; doi:10.1074/jbc.AC119.009198)
Supplement: Supporting Information [file supp_294_29_11054__index.html]

Liquid-liquid phase separation of tau protein: The crucial role of electrostatic interactions — Liquid-liquid phase separation of tau — Liquid–liquid phase separation of tau protein: The crucial role of electrostatic interactions — EDITORS' PICK: Liquid–liquid phase separation of tau — Supporting Information 

# Liquid–liquid phase separation of tau protein: The crucial role of electrostatic interactions

## Supporting Information

- Supporting Information (to be published online) - Supplementary Fig. S1
