# Supplementary material for: Liquid–liquid phase separation of tau protein: The crucial role of electrostatic interactions
Source: J Biol Chem. 2019 May 16;294(29):11054–9. doi: 10.1074/jbc.AC119.009198 (PMC6643045; doi:10.1074/jbc.AC119.009198)
Supplement: Supporting Information [file supp_AC119.009198_152604_1_supp_331138_prg109.pdf]

Supporting information for

Liquid-liquid phase separation of tau protein: The crucial role of electrostatic interactions

Solomiia Boyko[‡†], Xu Qi[‡], Tien-Hao Chen, Krystyna Surewicz, Witold K. Surewicz*

From the Deparment of Physiology and Biophysics, Case Western Reserve University, Cleveland, Ohio 44106, USA.

Running title: *Liquid-liquid phase separation of tau*

[‡]These authors contributed equally to this study
[†]Permanent address: Nencki Institute of Experimental Biology, Warsaw, Poland

*To whom correspondence should be addressed. Tel: 216-368-0139; Email: witold.surewicz@case.edu

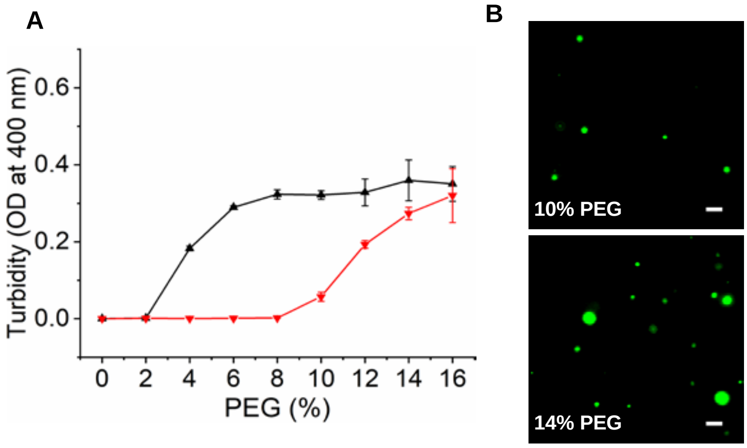

Figure S1. Phase separation of tau441 is not salt-specific. A, LLPS of tau441 (5 μM) monitored by turbidity as a function of PEG concentration. Black, 10 mM KCl; red, 150 mM KCl. Error bars represent SD (n=3). B, Fluorescence microscopy images of droplets formed by tau441 (5 μM) in the presence of 150 mM KCl at different PEG concentration. Scale bar, 3 μm.
